# The unequivocal preponderance of biocomputation in clinical virology

**DOI:** 10.1039/c8ra00888d

**Published:** 2018-05-18

**Authors:** Sechul Chun, Manikandan Muthu, Judy Gopal, Diby Paul, Doo Hwan Kim, Enkhtaivan Gansukh, Vimala Anthonydhason

**Affiliations:** Department of Environmental Health Science, Konkuk University Seoul 143-701 Korea; Environmental Microbiology, Department of Environmental Engineering, Konkuk University Seoul 143-701 Korea; Department of Biotechnology, Indian Institute of Technology-Madras Chennai 600036 India vimalalisha@gmail.com +91-44-22574102 +91-44-22574101

## Abstract

Bioinformatics and computer based data simulation and modeling are captivating biological research, delivering great results already and promising to deliver more. As biological research is a complex, intricate, diverse field, any available support is gladly taken. With recent outbreaks and epidemics, pathogens are a constant threat to the global economy and security. Virus related plagues are somehow the most difficult to handle. Biocomputation has provided appreciable help in resolving clinical virology related issues. This review, for the first time, surveys the current status of the role of computation in virus related research. Advances made in the fields of clinical virology, antiviral drug design, viral immunology and viral oncology, through input from biocomputation, have been discussed. The amount of progress made and the software platforms available are consolidated in this review. The limitations of computation based methods are presented. Finally, the challenges facing the future of biocomputation in clinical virology are speculated upon.

## Abstract 2

Biocomputation in clinical virology.

## Graphical abstract

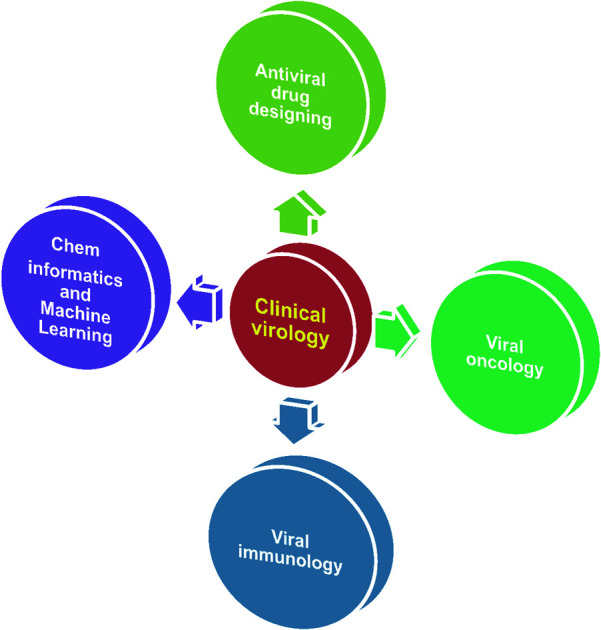

## 1. Introduction

Generally, viral outbreaks are pandemic and mostly transmitted *via* oral and nasal passages, the gastrointestinal tract, the skin and the urogenital tract/vagina. Viral diseases are not only a health threat, but they also result in outbreaks that have huge impacts on the global economy. Viral infections are a tremendous disease burden on humanity and combating them is an increasing challenge, since new viruses are continuously found. According to a recent World Health Organization (WHO) report, viral diseases which have recently caused outbreaks include: Ebola virus, influenza, human immunodeficiency virus (HIV), Middle East respiratory syndrome coronavirus (MERS-CoV), severe acute respiratory syndrome (SARS) and Zika viral infection.^1^ These diseases are major threats to health and global security. From 2013 to 2016, Ebola virus was most predominant in Guinea, Sierra Leone and Liberia. Since May 2016, 28 616 suspected cases have been found, 11 310 deaths reported and the fatality rate was approximately 70.8%.^2,3^ Outbreaks of Zika virus have been traced back to October 2015 in Brazil. The outbreak of Zika virus was evidenced by a rapid increase in cases among pregnant women, whose infants are born with extremely underdeveloped brains and will grow up to be adults with limited cognitive abilities and motor skills. The World Bank estimated that this outbreak caused a loss to the global economy to the magnitude of a total of $8.9 billion USD.^4^ The identification and characterization of the causative agents and prophylaxis to limit the spread of a virus require the successful isolation of viral isolates *via* ‘wet-lab’ experimentation. This is necessary for curbing these outbreaks, in order to facilitate restoration.

### 1.1 Importance of clinical virology

With morbidity and mortality rates being significantly high with respect to virus related infections, clinical virology is at the forefront of research highlights. Clinical virology is a field of medicine which consists of the identification of viral pathogens responsible for human diseases like polio, chikungunya, severe acute respiratory disease (SARS), influenza, acquired immune deficiency syndrome (AIDS), Ebola hemorrhagic fever, hepatitis *etc.*^5^ Clinical virology is an interdisciplinary field, integrating virology/medical virology and healthcare sciences that characterize the safety and efficacy of medication, devices, diagnostic products and treatment regimes intended for mankind, which can be utilized for the prevention, treatment and diagnosis of infectious viral diseases. The effective prevention and clinical management of infectious diseases are intimately linked to the early and accurate screening of pathogens. This includes detecting the infectious particles in the organism and elucidating the aspects that confer resistance to therapy, mutations and genotype disparity. In this aspect, the accurate interpretation of laboratory results warrants the effective clinical management and control of a disease,^6^ however on the other hand, erroneous diagnosis could lead to financial and human loss. EM and culture-based methods work together and were one of the early traditional methods for the diagnosis of viral infections, along with serology testing for the detection of antibodies targeted against viruses. These conventional methods are still fundamental practices in many medical labs.^7^ Cell culturing is yet another popular method for isolating viruses using cell lines.^8^ The complement fixation test (CFT) is one of the oldest methods in the history of clinical virology.^9^ The haemagglutination inhibition test is generally used for detecting arboviruses and influenza and parainfluenza virus subtypes and is capable of yielding relative quantitation of the virus particles.^10,11^ More recent, new generation diagnostic methods include: immunoassay methods,^12,13^ amplification based assays,^14,15^ mass spectrometric methods^16,17^ and next generation sequencing.^18,19^

Biocomputational tools and database resources provide a wealth of valuable information about viral genomic sequences, molecular structures and viral–host pathogenesis. This information on infectious agents can lead to better diagnostics, therapeutics and vaccine development. Bioinformatics addresses these specific needs during data acquisition, storage and analysis and for the integration of this research with major therapeutic research areas such as viral oncology, viral immunology and antiviral research. In this review, the importance of exploring the role of biocomputation for additional knowledge in the realms of clinical virology is discussed. Advances made in the fields of clinical virology, antiviral drug design, viral immunology and viral oncology, through input from biocomputation, are recorded. Computation based approaches, their effects on clinical virology and their therapeutic usefulness are presented. Finally, the challenges facing the future of biocomputation in clinical virology are speculated upon.

## 2. Current status of biocomputational approaches in clinical virology

**Fig. 1:**
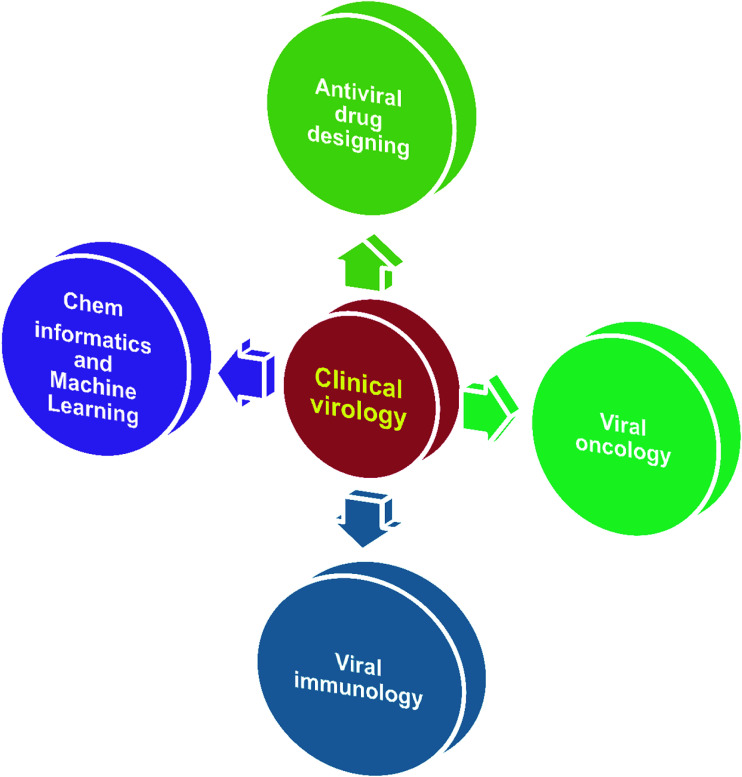
Scheme depicting the ongoing major research areas in clinical virology.

In virology, computational approaches have played crucial roles in various aspects of viral genome sequence analysis, therapeutic protein identification, anti-viral drug design/discovery, *in silico* vaccine design, differentially expressed gene identification, microRNA based signature identification and therapeutic design. Through *in silico* analysis, researchers gain a profound understanding of viral–host pathogenesis, which leads to better diagnostics, therapeutics and vaccines. Viruses have always been a major cause of a large number of infectious diseases. Molecular knowledge on viral proteins is thus seen to play an important role in the development of improved peptide-based vaccines, the design of novel anti-viral agents and the understanding of the entry mechanisms of viruses. Ongoing major research areas where biocomputation has played a positive role in clinical virology are represented in Fig. 1.

### 2.1 Biocomputation for antiviral drug design

Antiviral drugs are aimed at targeting selective viral infections. In particular, antiviral drugs have been designed against herpes, HIV, human cytomegalovirus (HCMV), varicella-zoster virus (VZV), hepatitis B and C, and influenza A and B viral infections which have caused chronic infection in millions all over the world.^20^ There are many difficulties facing the accurate design of an antiviral drug against pathogenic viruses. Since viruses are parasites, they are unable to replicate on their own. They reproduce within the cells of an infected host and disturb the functions of the host cells. Many clinically important viruses don’t have model systems due to their dangerous ability to mutate. Cultures are also hard to maintain and expensive. Viruses which show early infectious symptoms like influenza, common cold *etc.*, are treated with antiviral drugs, but for viruses with symptoms that appear during the later stages of infection, the population is put at risk.^21^

A very early stage of viral infection is viral entry; a second approach is to target the processes that synthesize viral components after a virus invades a cell and establishes a critical infection. Based on the level of infection, the receptor protein and its therapeutic compounds are different.^22^ So before designing a drug, we need to ascertain the level of infection. To date, however, many viruses remain devoid of effective immunization and only a few antiviral drugs are licensed for clinical practice. Hence, there is an urgent need to discover novel antivirals that are highly efficacious and cost-effective for the management and control of viral infections at times when vaccines and standard therapies are lacking.^23^

Previously identified antiviral drugs do not destroy the target pathogen of the host, but instead they are able to reduce their growth and development. Modern *in silico* antiviral drug design aims to identify viral proteins (receptor proteins) that induce disease in humans or the survival of a microbial pathogen. Target proteins are common among many strains of viruses. Once the potent targets are identified, the appropriate drugs can be designed and administered.^24^ Target protein sequences were retrieved from sequence databases like viral genome databases (see Table 1) and structures will be retrieved from viral protein databases (Table 1). The binding site of the receptor protein is called the active site and this will be identified using *in silico* binding site prediction tools, such as active site prediction servers, CASTp,^36^ PDBeMotif,^37^ metaPocket,^38^ 3DLigandSite,^39^ Pocketome,^40^ PocketDepth,^41^ Pocket-Finder,^42^ FINDSITE^43^*etc.* Drugs can be designed which bind to the active region and inhibit receptor molecules. The structure of a drug molecule that can specifically interact with the biomolecules can be modeled using molecular modeling techniques *via* computational tools like I-TASSER,^44^ Robetta,^45^ HHpred,^46^ MODELLER,^47^ MODBASE,^48^ RaptorX,^49^ SWISS-MODEL^50^*etc.*

**Table 1:** A consolidated list of various biocomputational resources used in clinical virology

Feature	Computational resource	Website	Reference
Viral sequence database	Viral genomes resource	https://www.ncbi.nlm.nih.gov/genome/viruses/	25
	Virus pathogen database and analysis resource (ViPR)	https://www.viprbrc.org/	26
	Viral genome databases (VGDB) – Oxford academic	https://www.oxfordjournals.org/nar/database/subcat/5/18	27
	Viral reference sequences – ViralZone	http://www.viralzone.expasy.org/6096	28
	Influenza research database	https://www.fludb.org/	29
	viruSITE	http://www.virusite.org/	30
	RNAVirusDB	http://virus.zoo.ox.ac.uk/rnavirusdb	31
		http://hivweb.sanbi.ac.za/rnavirusdb	
		http://bioinf.cs.auckland.ac.nz/rnavirusdb	
		http://tree.bio.ed.ac.uk/rnavirusdb	
Viral protein structure database	VPDB	http://vpdb.bicpu.edu.in/	32
	VIDA	http://www.biochem.ucl.ac.uk/bsm/virus_database	33
	VIPERdb	http://viperdb.scripps.edu/	34
	PhEVER	http://pbil.univ-lyon1.fr/databases/phever	35
Antiviral drug database	Antiviral library	http://www.enamine.net/	101 and 102
	Drug office – oral antiviral drugs	https://www.drugoffice.gov.hk/eps/do/en/consumer/news_informations/dm_17.html	103
	Virus pathogen database and analysis resource (ViPR)	https://www.viprbrc.org/	26
	Database of anticancer peptides and proteins (CancerPPD)	http://crdd.osdd.net/raghava/cancerppd/	104
	Cancer drug resistance database (CancerDR)	http://crdd.osdd.net/raghava/cancerdr/	105
	CancerHSP	http://lsp.nwsuaf.edu.cn/CancerHSP.php	106
		http://www.inpacdb.org	107
Microarray data	Stanford microarray database (SMD)	http://genome-www.stanford.edu/microarray/	108
	GEO datasets	https://www.ncbi.nlm.nih.gov/geo/info/datasets.html	109

**Fig. 2:**
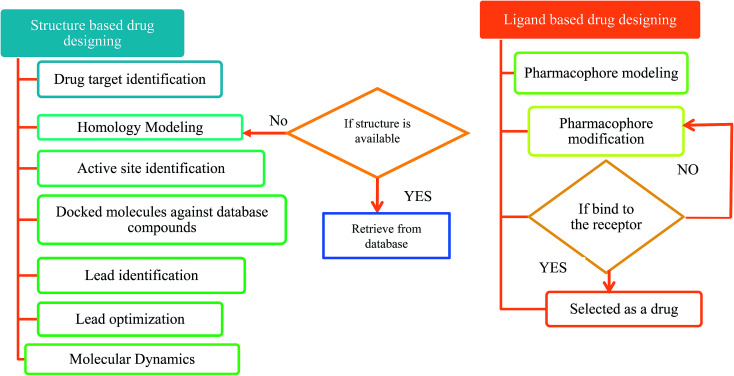
A flowchart for antiviral drug design.

The first antiviral drug 5-iodo-2′-deoxyuridine (idoxuridine, IDU) was designed for the herpes virus infection in 1960.^51^ Computer aided drug design (CADD) played an important role in designing a suitable antiviral drug against the deadly viral infection. There are two types of CADD, structure based and ligand based.^52^ The available drugs were retrieved from antiviral drug databases (see Table 1). The successful design of antiviral drugs like Saquinavir, Relenza, and Tamiflu has validated the application of these techniques and indicates a bright future for drug discovery protocols.^53^ The creation of a structure based pharmacophore model from the 3D structure of a target protein provides helpful information for investigating protein–ligand interactions and upgrading the knowledge of ligand binding affinities. The structure based pharmacophore regions are used for screening chemical databases for potential lead structures. CATALYST,^54^ PharmDock,^55^ PHASE,^56^ and ROCS^57^ are used for pharmacophore modeling. Molecular docking is used to study the interactions between a target protein and a ligand molecule. Tools and software used for molecular docking include: AutoDock,^58^ DOCK,^59^ Glide,^60^ GOLD,^61^ PatchDock^62^*etc.* Molecular dynamics (MD) simulations can be used to provide dynamic insight into the structures of viruses and their components.^63^ These simulations can be performed using CHARMM,^64^ Desmond,^65^ GROMACS^66^*etc.* A flowchart for a typical antiviral drug design process is shown in Fig. 2.

### 2.2 Biocomputation in viral immunology and subsequent *in silico* vaccine design

Viral immunology is the most challenging and rapidly growing field in biology. During computational approaches, it relates to computational immunology or immunoinformatics, which deal with immunological problems using computational methods.^67^ Generally, the immune system connects thousands of molecules which become closely and intricately linked with each other; based on individuals the structure and the function of these molecules are different. Experimentally, immunologists have generated a vast amount of functional, clinical and epidemiological data. Using *in silico* techniques, these data have been stored and analyzed. *In silico* methods are affordable, because they reduce the time and costs involved in the laboratory analysis of pathogenic proteins. *In vivo* methods require the cultivation of the pathogenic genome in order to identify antigenic proteins. Although pathogens grow quickly, the extraction of their proteins and then the testing of those proteins on a large scale is expensive and time consuming.^68^ The use of immunoinformatics solves these problems and can identify virulence genes, potential target proteins and binding sites; this will lead to the development of novel vaccines and immunotherapeutics. This *in silico* method for vaccine design is called reverse vaccinology.

In 1796, Edward Jenner first identified the smallpox vaccine and introduced the term vaccine.^69^ A century later, Louis Pasteur introduced a rational method for vaccine development.^70^ Subsequently, researchers discovered many *in vivo* vaccines like the polio vaccine (Albert Sabin),^71^ measles, mumps and rubella (Hilleman),^72^ and so on. In later years, researchers faced the challenge of cultivating pathogenic viruses. It was found that in the case of hepatitis B viral infection, the pathogenic virus could not be cultured *in vitro*. Thus, they collected the viral antigen from chronically infected patients, the antigens were inactivated and the vaccine was developed.^73^ At the end of the 20^th^ century, researchers developed vaccines based on the genomes of microorganisms. The first pathogenic organism identified by the reverse vaccinology approach was meningococcus B.^74^ Recently, Reza Taherkhani and Fatemeh Farshadpour developed an epitope based vaccine for the hepatitis E viral infection^75^ and vaccines have been designed for other recent outbreaks including for the Zika virus^76^ and the Ebola virus.^77^

**Fig. 3:**
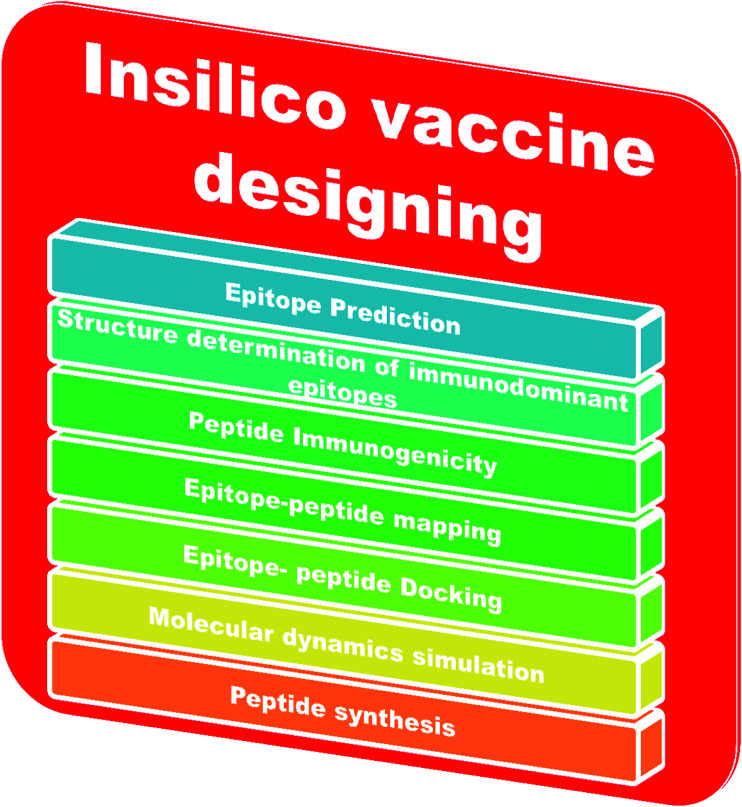
Steps in *in silico* vaccine design.

An adaptive immune response occurs against a pathogen. There are two types of adaptive immune response, cellular and humoral, mediated by T cells and B cells. The antigenic determinant region is called the epitope, which is recognized by the corresponding receptor present on B or T cells.^78^ In bioinformatics, epitope based vaccine design relies on machine learning techniques, such as support vector machines (SVMs), hidden Markov models (HMMs) and neural networks (NNs).^79–81^ The *in silico* vaccine design process requires the identification of a key molecule on the receptor protein, which is predicted using the Epitope Database (IEDB).^82^ Then, the immunogenetic peptides will be identified using Kolaskar and Tongaonkar’s predicted antigenic peptide tools, based on applied semi empirical methods,^83^ and the peptide-epitope will be mapped. Subsequently, peptide and immunodominant epitope docking will be performed for vaccine design. Molecular dynamics based simulations will be carried out for conformational analysis. Finally, the peptide will be synthesized for practical application. The steps involved in *in silico* vaccine design are shown in Fig. 3.

### 2.3 Biocomputational inputs in viral oncology

Viral oncology is an ongoing and essential research field for biologists. According to a report in 2017 by the American Cancer Society, 1 688 780 cancer cases were expected to be treated in 2017.^84^ Certain tumors are reported to be caused by viruses like the human papilloma virus (HPV), Epstein–Barr virus (EBV), Kaposi’s sarcoma-associated herpesvirus (KSHV), hepatitis C virus (HCV), human immunodeficiency virus (HIV) and hepatitis B virus (HBV). More than 90% of anal cancers have been caused by 500 000 HPV infections per year worldwide.^85^ Traditionally, viral oncology research has relied on biological techniques, but recently, computational techniques have enabled the diagnosis of malignancies.

**Fig. 4:**
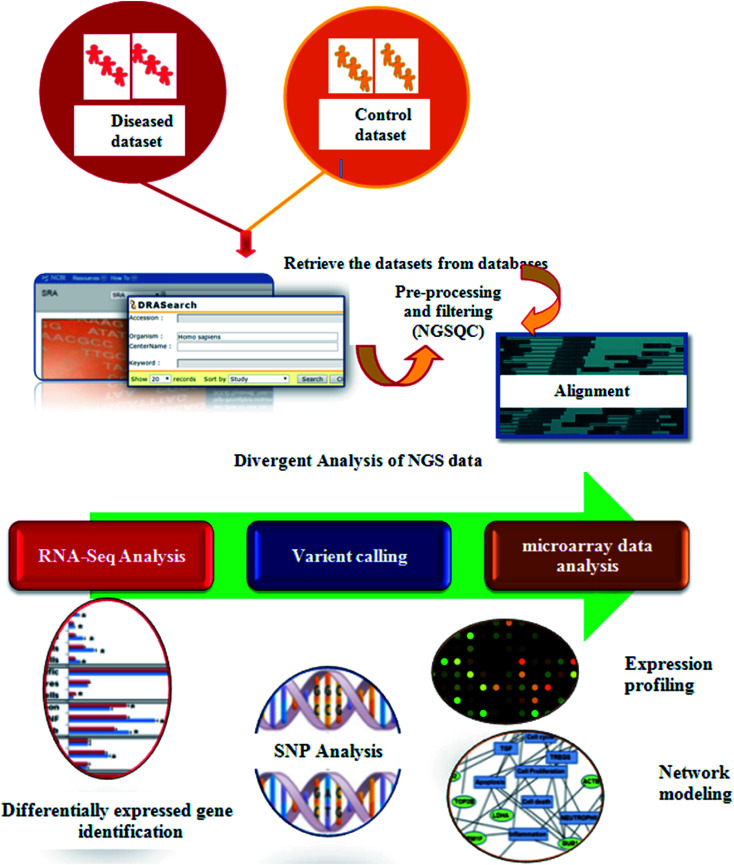
Schematic representation of the process steps of the NGS of clinical data.

In recent years, high throughput technologies have generated vast amounts of data, but on the other hand only limited experimental information is available for most genes. Next Generation Sequencing (NGS) plays an especially important role in cancer therapeutics. Computers can analyze vast amounts of data using remarkable techniques based on supercomputers. Computational oncology techniques have created *in silico* biological system models. To understand normal *vs.* malignant patient sequence characteristics, bioinformaticians first retrieved enormous sequences from various databases.^86^ Tumor suppressor genes (TSG), sequence annotation, their relation to diseases and gene ontology (GO) processes are identified using these databases. Cellular pathway databases have played an important role in identifying the essential proteins that induce disease, constructing biological networks and predicting models.^87^ Using traditional methods, only single genotype–phenotype relationships can be identified at a time. In contrast, high-throughput technology examines the phenotypic outcomes of multiple mutations simultaneously.^88^Fig. 4 presents the typical process steps of the NGS of clinical data.

Microarrays contain thousands of gene expressions and transcriptome data. Using a computer, we can analyse microarray data. From transcriptome data, differentially expressed genes can be identified using NGS techniques. The differentially expressed genes lead to the identification of cellular target genes that identify the therapeutic compound.^89,90^ Computer based drug design techniques use the pharmacokinetics and pharmacodynamic relationships of the available anticancer agents to improve treatment and drug development. These techniques include virtual screening, QSAR (quantitative structure activity relationship) models and molecular docking.^91^ Pharmacophore-mapping algorithms are employed for the inverse screening of some representative compounds for a large set of pharmacophore models constructed from human target proteins. Molecular docking studies were carried out to assess the binding affinity of these compounds to proteins responsible for mediating tumor growth. Furthermore, the important structural features of compounds for anticancer activity were assessed using Monte Carlo-based SMILES and hydrogen graph-based QSAR studies.^92^

### 2.4 Cheminformatics and machine learning models for anti-viral drug discovery

Structure activity relationships (SARs) with molecular systems, quantitative structure activity relationships (QSARs) and quantitative structure–property relationships (QSPRs) with molecules play a major role in computational drug discovery. In this context, machine learning approaches for viral drug discovery are pioneering as an emerging and ongoing research technique in clinical virology. Machine learning approaches can help make complex network models for confirming the effectiveness of combinations of drugs for epidemiological outbreaks in large populations. In 2015, Herrera and his coworkers collected datasets from ChEMBL (anti-HIV chemical compounds), the AIDSVu database (HIV surveillance reports) and the Census Bureau (socioeconomic data) and proposed the first artificial neural network (ANN) model for the prediction of HAART cocktails, to halt AIDS in the epidemic networks of the U.S.^93^ The first multitasking model for quantitative structure–biological effect relationships (mtk-QSBERs) was predicted, *in silico* fragment based drug design for drug–molecule interaction study was performed and its molecular entities were screened by virtual screening for the Hepatitis C viral infection. This was then experimentally validated for anti-HCV activity and ADMET (Absorption, Distribution, Metabolism, Excretion and Toxicity) properties.^94^ Very recently, the first multitasking model was developed for anti-HIV agents from 29 682 HIV cases using quantitative structure–biological effect relationships (mtk-QSBER) based on fragment based drug design and virtual screening approaches. More than 96% of fragments contributed towards the multiple biological effects.^95^

ANNs have been used to link data related to AIDS in the U.S. to ChEMBL data. ANNs are network prediction models that are mainly used in medicinal chemistry and drug development.^96^ Moreover, in 2008 Francisco and coworkers constructed drug–drug complex networks against different species of virus.^97^ González-Díaz *et al.*, used the MARkovian CHemicals *IN SIlico* DEsign (MARCH-INSIDE) approach and predicted novel antimicrobial drugs and targets using drug–drug similarity complex networks.^98^ Computational studies of structural stability relationships produce novel stochastic moments. In 2005, González-Díaz *et al.* constructed a new Markov model, which makes use of novel stochastic moments such as molecular descriptors for viral protein surfaces in quantitative structure–activity relationship (QSAR) studies for small molecules for human rhinoviruses (HRVs).^99^ Markovian Backbone Negentropies (MBNs) have been introduced in order to model their effects on protein structure stability relationships. An MBN based on a Markov chain model of electron delocalization throughout the protein backbone for the computational study of structure/stability relationships has also been reported.^100^ These approaches mainly focus on cheminformatic approaches towards drug design, drug development and drug–target interactions in *in silico* clinical fields, especially with respect to viral diseases.

### 2.5 Challenges facing biocomputation in clinical virology

Antiviral drug discovery and design processes are not exceptionally without fault; they do have certain limitations and face a few challenges. Firstly, therapeutic target selection is difficult in certain conditions, for instance in the disordered physiological processes (pathophysiology) of nervous system related disorders. Thus, the integration of large experimental data with machine learning approaches requires the development of new brain-inspired computational algorithms.^110^ If the molecular mechanisms of the disease are unknown, it is difficult to find the specific receptor and with the function of the protein not determined, it is difficult to make much progress.^111^ No single medicine is a common solution for all diseased patients; it may vary based on the patient’s symptoms, disease conditions and their history. In such a case, we have to follow a personalized medicinal approach. Based on this approach we need gene expression data for individuals in order to identify the potent drug target and design a drug and in such situations we need a knowledge base. We need to investigate many drug targets based on the drug candidate, which is a trial and error process.^112^ The use of molecular signatures like peptides in epitope based vaccine design hits a limitation here due to a lack of delivery systems or disease models, rendering it incapable of reaching its set goals in therapeutics.^113^ The mtk-QSBER model was able to integrate multiple chemical compounds with multiple biological target molecules and develop drug like compounds.^93,94,114–116^

Emerging diseases, especially viral diseases including cancer related viruses, threaten human life with mortality and morbidity. Emerging viral diseases caused by RNA viruses are increasing due to increasing mutation rates. In clinical virology, emerging techniques, such as microarrays (expression analysis), metagenomic biosynthetic gene cluster identification, host–pathogen interaction analysis, unknown disease-associated viruses, the discovery of novel human viruses and NGS technologies (differential gene expression, *de novo* sequencing, epigenetics, variant calling, SNPs *etc.*), are enormously supported.^117,118^ Using these technologies, it is possible to analyze millions of data simultaneously at a low cost.

**Fig. 5:**
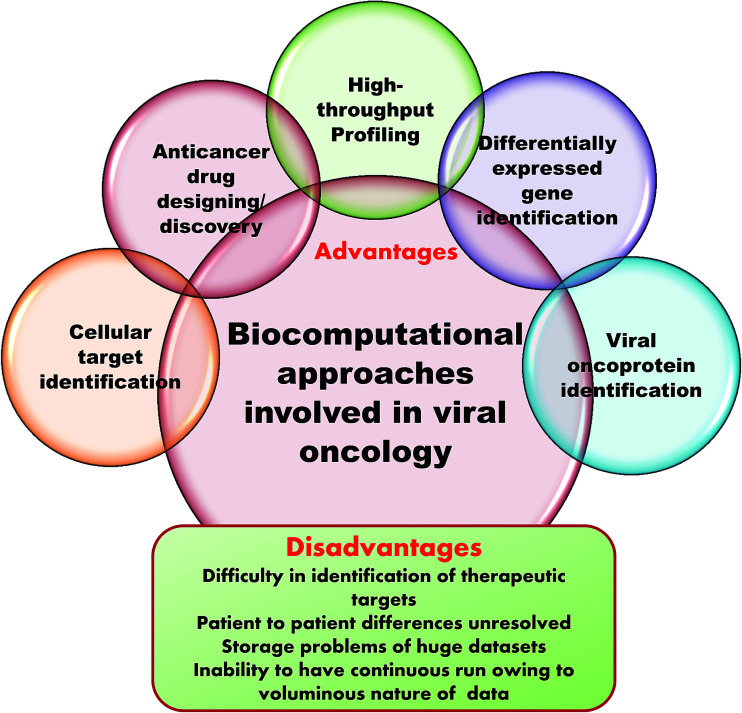
Advantages and disadvantages of biocomputation in clinical virology.

Microarrays generate a vast amount of gene expression datasets for healthy and control samples. NGS also produces gigabytes of transcriptome data. Here, the major challenge is storage of this voluminous data. This requires multiprocessors and multicore computers with hundreds of gigabytes of RAM and terabytes of hard drive space in Linux operating systems. Even with the use of internet based supercomputing, the delivery of results is greatly delayed and results become queued up. It is also impossible to submit whole datasets as these have to be divided up to adhere to the accessible memory size and then submitted. In this way, the entire process is greatly affected and becomes time consuming because of the data storage issue.^119^Fig. 5 summarizes the advantages and disadvantages of computation in clinical virology.

## 3. Summary

Computer-assisted clinical virology research areas are without doubt very essential, although many aspects of improvement are still in progress. Every year, lots of web-assisted tools, software and algorithms are continuously being developed for various aspects of antiviral drug design, viral immunology and viral oncology. High throughput technologies generate data for thousands of patients as well as their corresponding expression data. Thus, computer-assisted approaches do overcome cumbersome wet lab procedures to a large extent and provide valuable insights into clinical virology and a positive direction for antiviral drug design, epitope-based vaccine design, differentially expressed gene identification and potential drug target identification strategies.

## Conflicts of interest

The authors have no conflicts of interest whatsoever.

## Supplementary Material
